# Data on isolating mesenchymal stromal cells from human adipose tissue using a collagenase-free method

**DOI:** 10.1016/j.dib.2016.02.002

**Published:** 2016-02-08

**Authors:** Wassim Shebaby, Eddie K. Abdalla, Fady Saad, Wissam H. Faour

**Affiliations:** a Department of Natural Sciences, School of Arts & Sciences, Lebanese American University, Byblos, Lebanon, P.O. Box 36, Lebanon; b Lebanese American University Medical Center – Rizk Hospital, Beirut, Lebanon; c Saint Louis Hospital, Jounieh, Lebanon; d School of Medicine, Lebanese American University, Byblos, Lebanon, P.O. Box 36, Lebanon

**Keywords:** Adipose tissue, mesenchymal stromal cell, cell culture, doubling time

## Abstract

The present dataset describes a detailed protocol to isolate mesenchymal cells from human fat without the use of collagenase. Human fat specimen, surgically cleaned from non-fat tissues (e.g., blood vessels) and reduced into smaller fat pieces of around 1–3 mm size, is incubated in complete culture media for five to seven days. Then, cells started to spread out from the fat explants and to grow in cultures according to an exponential pattern. Our data showed that primary mesenchymal cells presenting heterogeneous morphology start to acquire more homogenous fibroblastic-like shape when cultured for longer duration or when subcultured into new flasks. Cell isolation efficiency as well as cell doubling time were also calculated throughout the culturing experimentations and illustrated in a separate figure thereafter. This paper contains data previously considered as an alternative protocol to isolate adipose-derived mesenchymal stem cell published in “*Proliferation and differentiation of human adipose-derived mesenchymal stem cells (ASCs) into osteoblastic lineage are passage dependent”* [1].

## 1 Specifications Table

**Table:** 

Subject area	*Cell Biology*
More specific subject area	*Cell culture, mesenchymal stromal cells*
Type of data	*Image (microscopy), text file, graph, figure*
How data was acquired	*Cell counting using light microscope, statistical analysis, math formula calculation*
Data format	*Raw and analyzed data*
Experimental factors	*Cells isolated from fat explants cultured in plastic flasks*
Experimental features	*Spontaneous isolation of mesenchymal cell using in vitro cell culture system and without the use of collagenase*
Data source location	*Lebanese American University, Byblos, Lebanon*
Data accessibility	*Data are provided in the article*

## 2 Value of the data

• The below data provide a detailed and reproducible collagenase-free protocol to isolate mesenchymal stromal cells from human adipose tissue.
• These data enable researchers to isolate various cell types populating fat.
• These data offer the possibility to isolate specific primary cell cultures with a reduced and efficient cell isolation yield.

## 3 Data

**Fig. 1:**
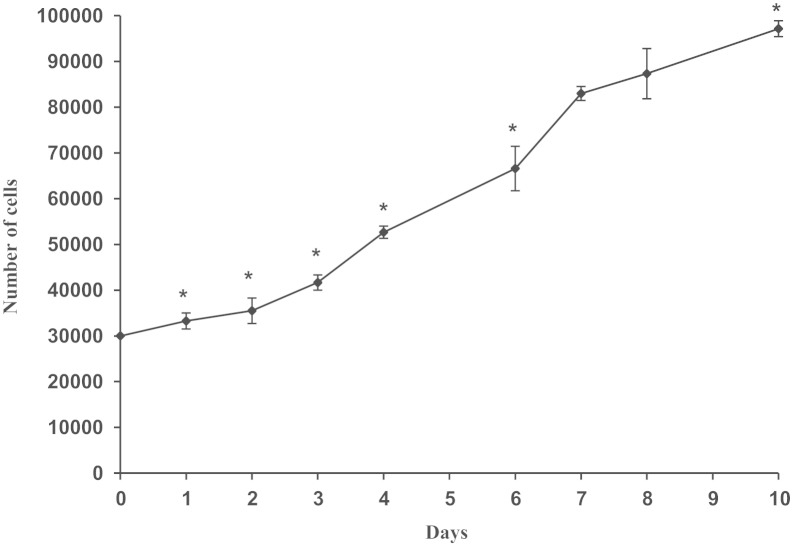
Growth kinetic results of adherent mesenchymal cells at passage 1. Primary mesenchymal cells were trypsinized and seeded in 6-well plates at a density of 3000 cells/cm^2^ rendering cells at passage 1. Cell number in each well was determined with trypan blue exclusion count in triplicate as indicated in methods. Growth rate is compared between various cultivation times. Error bars represent the standard deviation. Values denote mean (*n*=3), ^*^P<0.05 with respect to day 7.

**Fig. 2:**
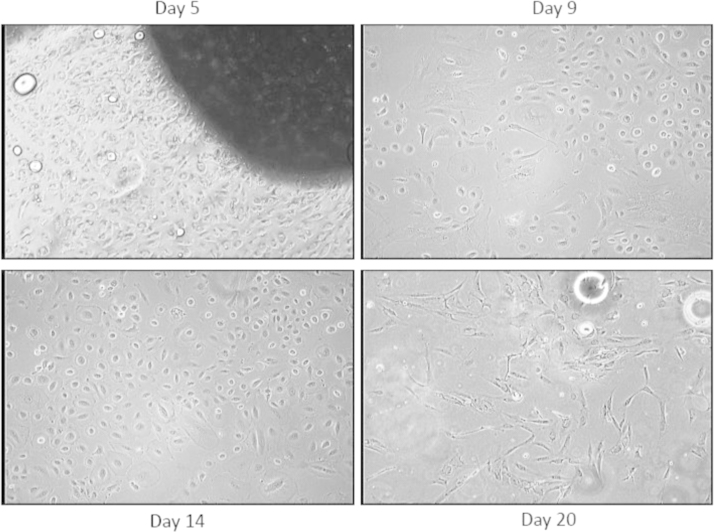
Morphology of primary mesenchymal cells at various cultivation times. Primary mesenchymal cells spreading from fat explants were cultured in T75cm^2^ culture plate until confluency as indicated. Morphologic appearance of mesenchymal cells was shown at 10× light microscopy at different times for a maximum of 20. Fat explants were removed from the culture medium at day five to seven.

**Fig. 3:**
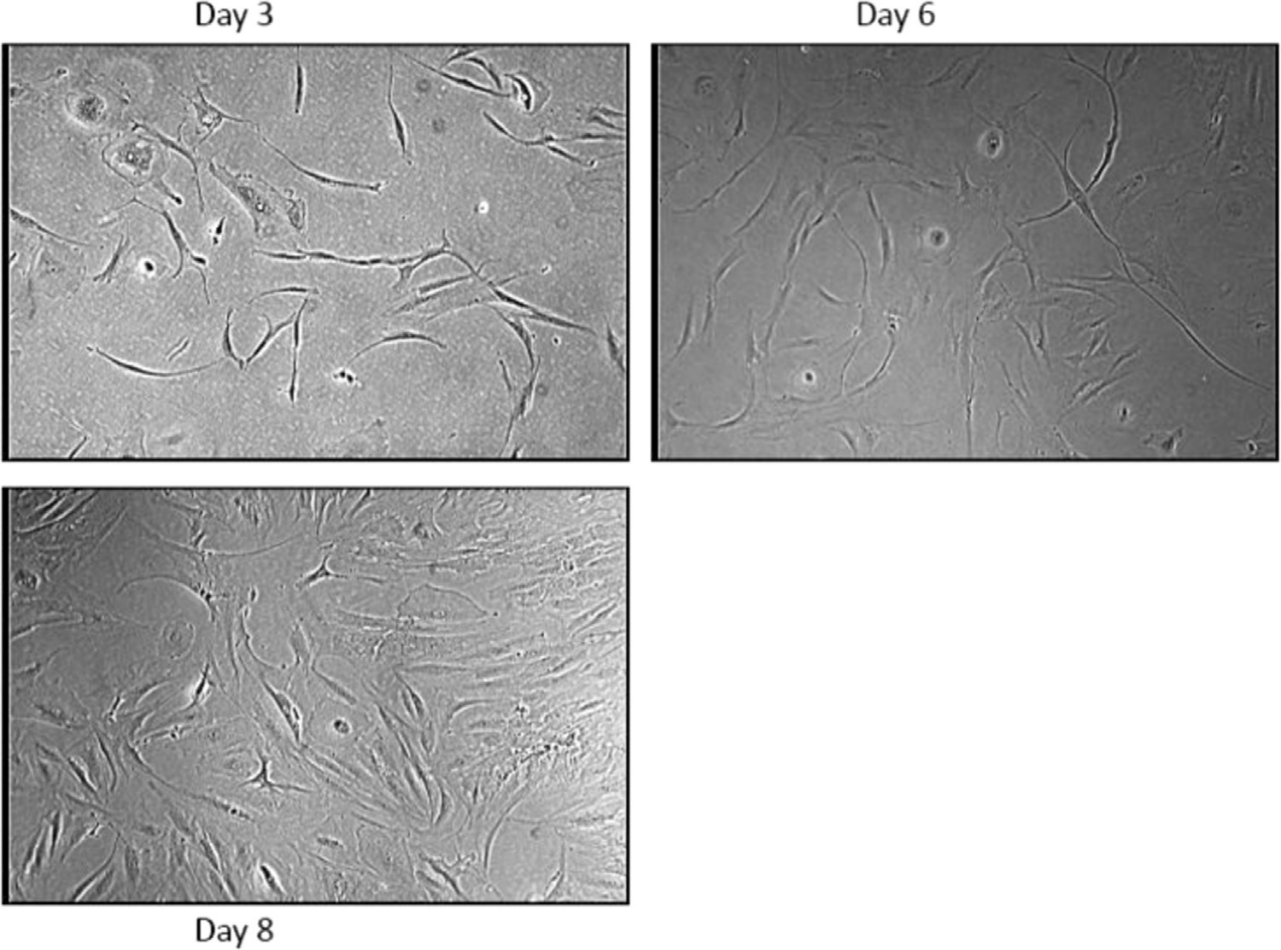
Morphology of mesenchymal cells at passage 1 at various cultivation times. Subcultivated (trypsinized) mesenchymal cells seeded at a density of 3000 cells/cm^2^ were cultured until confluency as indicated. Morphologic appearance of mesenchymal cells was shown at 10× light microscopy at different cultivation times until confluency and/or quiescence for a maximum of 10 days.

The data presented in this paper correspond to the isolation of mesenchymal stromal cells without digesting human fat pieces with collagenase. Subsequently, cell morphology, primary cell isolation efficiency as well as cell population doubling time were measured using light microscopy analysis and mathematical formulas. These data were considered as an alternative method to isolate stem cells from human adipose tissues in our previously published manuscript [1] (Fig. 1, Fig. 2, Fig. 3).

## 4 Experimental design, materials and methods

### 4.1 Technical procedure

#### 4.1.1 Fat specimen

Fresh fat specimen obtained from surgery is incubated in a sterile saline solution at room temperature and processed within 2–4 h postsurgery. Procedures were approved by the Institutional Review Board (IRB) of the Lebanese American University. Patients were chosen not to have chronic diseases or cancers. Abdominal adipose tissue resected from middle aged men patients aged 40–50 years old and were asked to read the consent forms and approve/sign them prior to surgery. Primary cells isolated from different donors were mixed together and then subcultured according to the protocol described below.

#### 4.1.2 Protocol of primary cell isolation and culture

Five grams of human fat are incubated in 40 ml of warm sterile PBS (1×) containing 3% Pen/Strep for 30 min. Then extra non-fat tissue (e.g., blood vessels and connective tissues...) were surgically removed under sterile conditions. One gram of tissue is minced into pieces of 8–10 mm^3^ under sterile conditions. Minced fat pieces were equally distributed into two T70cm^2^ cell culture plates (0.5 g of fat/plate) containing each 5 ml of complete growth medium [RPMI+10% FBS+1% penicillin/streptomycin (100 U/ml penicillin, 100 µg/ml streptomycin)] and incubated in CO_2_ cell culture incubator at 37 °C and left undisturbed for 3 days. After 3 days of culture the growth media is replaced with a new fresh growth media every 3 days by gently bending the plate and aspirating the old media with a sterile pipet. On day six the explants cultures can be observed under light microscopy and cells appear to spread out of the fat pieces into the surroundings of the fat specimens. At day 9, fat pieces are removed with a sterile pipet and the cell cultures were then washed twice with 5 ml of sterile warm PBS (1×) and finally cultured with 10 ml of new fresh complete growth medium until near confluency for further experimental analysis.

#### 4.1.3 Cell splitting

Cell splitting is done using a near confluent plate. First the plate is washed twice with 5 ml of sterile warm PBS (1×), then 3 ml of warm sterile trypsin (1×) are added, after discarding the wash buffer, and the plate is incubated in a CO_2_ cell culture incubator at 37 °C for 2–3 min. After adding 10 ml of complete growth media to inhibit trypsin effect, the medium is collected and transferred into sterile 50 ml conical tube, the remaining cells in the plate can be collected by an additional washing with 2 ml of sterile warm PBS (1×). The cells are separated by centrifugation at 2000 RPM for 5 min at room temperature. The supernatant is discarded while the pellet is resuspended with the desired volume complete fresh growth medium. Finally, cells are counted using a hemocytometer and appropriate number of cells are seeded as desired for the experimentation.

### 4.2 Data calculation

#### 4.2.1 Primary cell isolation efficiency (CIE) calculation

To measure the primary cell isolation efficiency (CIE) of cells isolated from fat explants, primary cells remaining after removing fat and subsequent wash are trypsinized, manually counted on a hemocytometer with trypan blue exclusion method and cell stained with trypan were excluded from the count. Then, CIE is calculated according to the following mathematical formula: CIE=[(number of primary cells obtained immediately after removing fat pieces)/(number of primary days in culture)]/(total mass of fat explant in grams). Number of primary cells obtained immediately after removing fat pieces=3×10^5^; number of primary culture days=9; total mass of fat explant=0.5 g. CIE=66,000/gram tissue.

#### 4.2.2 Growth kinetic protocol and calculation of cell population doubling time

To measure growth kinetic of cell cultures isolated from explants, primary mesenchymal cells remained after removing fat pieces and subsequent wash, are trypsinized, manually counted on a hemocytometer with trypan blue exclusion method and cells stained with trypan were excluded from the count. Trypsinized primary cells were plated at a density of 3×10^3^ cells per cm^2^ in 6-well plates to make a total of 30 wells. Cells were then kept overnight to adhere and maintained for ten days with medium changes occurring every two days starting the day after plating (day 0) and continuing each day until day 9. Three dishes were trypsinized every day to determine cell number and viability using trypan blue exclusion methods and cells stained blue with trypan were excluded from the count. Cell population doubling time (DT) of cultured mesenchymal cells was calculated using the following formula: DT=(*t*×Ln 2)/(Ln N2−Ln N1), *t*=number of days in culture, N1=number of cells at the beginning of the log phase, N2=number of cells at the end of the log phase. N1=35.500±2784 correspond to the number of cells at day 2, and N2=83,000±1528 correspond to the number of cells at day 7, then DT=4.12±0.4.

### 4.3 Statistics

All numerical data are presented as mean±standard error. Statistics analysis is done using *t* test. *P* value of less than 0.05 was considered to be statistically significant. Differences were evaluated by one-way analysis of variance (ANOVA). Differences were considered significant at (*P<0.05). All experiments were done in triplicate and data were expressed as mean±SEM.
